# Skin lesion and mortality rate estimates for common bottlenose dolphin (*Tursiops truncatus*) in the Florida Panhandle following a historic flood

**DOI:** 10.1371/journal.pone.0257526

**Published:** 2021-10-07

**Authors:** Christina N. Toms, Tori Stone, Traci Och

**Affiliations:** 1 Chicago Zoological Society’s Sarasota Dolphin Research Program, c/o Mote Marine Laboratory, Sarasota, Florida, United States of America; 2 Department of Biology, University of Central Florida, Orlando, Florida, United States of America; 3 Center for Environmental Diagnostics and Bioremediation, University of West Florida, Pensacola, Florida, United States of America; 4 Department of Biology, University of West Florida, Pensacola, Florida, United States of America; Universidad de los Andes, COLOMBIA

## Abstract

Increasing evidence links prolonged freshwater exposure to adverse health conditions, immune deficiencies, and mortality in delphinids. Pensacola, Florida, experienced a record-breaking flood event in April 2014, after which, skin lesions evident of freshwater exposure were observed on common bottlenose dolphins (*Tursiops truncatus*). Here we assess the potential consequences of the flood on bottlenose dolphin health and mortality. Data from an ongoing study were used to evaluate the relationship between skin lesions (progression, prevalence, and extent) and the flood with respect to changing environmental conditions (salinity). Annual stranding records (2012–2016) from Alabama to the eastern Florida Panhandle were used as an indicator of dolphin health to test the hypothesis that the flood event resulted in increased annual mortality rates. Although salinities remained low for several months, results suggest that there was not the widespread skin lesion outbreak anticipated. Of the 333 unique individuals detected only 20% were seen with skin lesions. There was a significant increase in the proportion of dolphins seen post-flood with lesion extent above background levels (≥ 5%; *p* = 0.001), however, there were only 11 cases with lesion extent greater than 20%. Skin lesion prevalence increased overall following the flood (*p* < 0.001), but pairwise comparisons revealed a delayed response with significant increases not detected until the following fall (*p* = 0.01), several months after salinities returned to expected levels. Regression modeling revealed no significant effects of year, region, or year x region on mortality rates, except in Alabama, where increased mortality rates were likely due to residual impacts from the Deepwater Horizon Oil Spill. This study takes advantage of a natural experiment, highlighting how little is understood about the conditions in which prolonged freshwater exposure leads to negative impacts on dolphin health.

## Introduction

**Fig 1:**
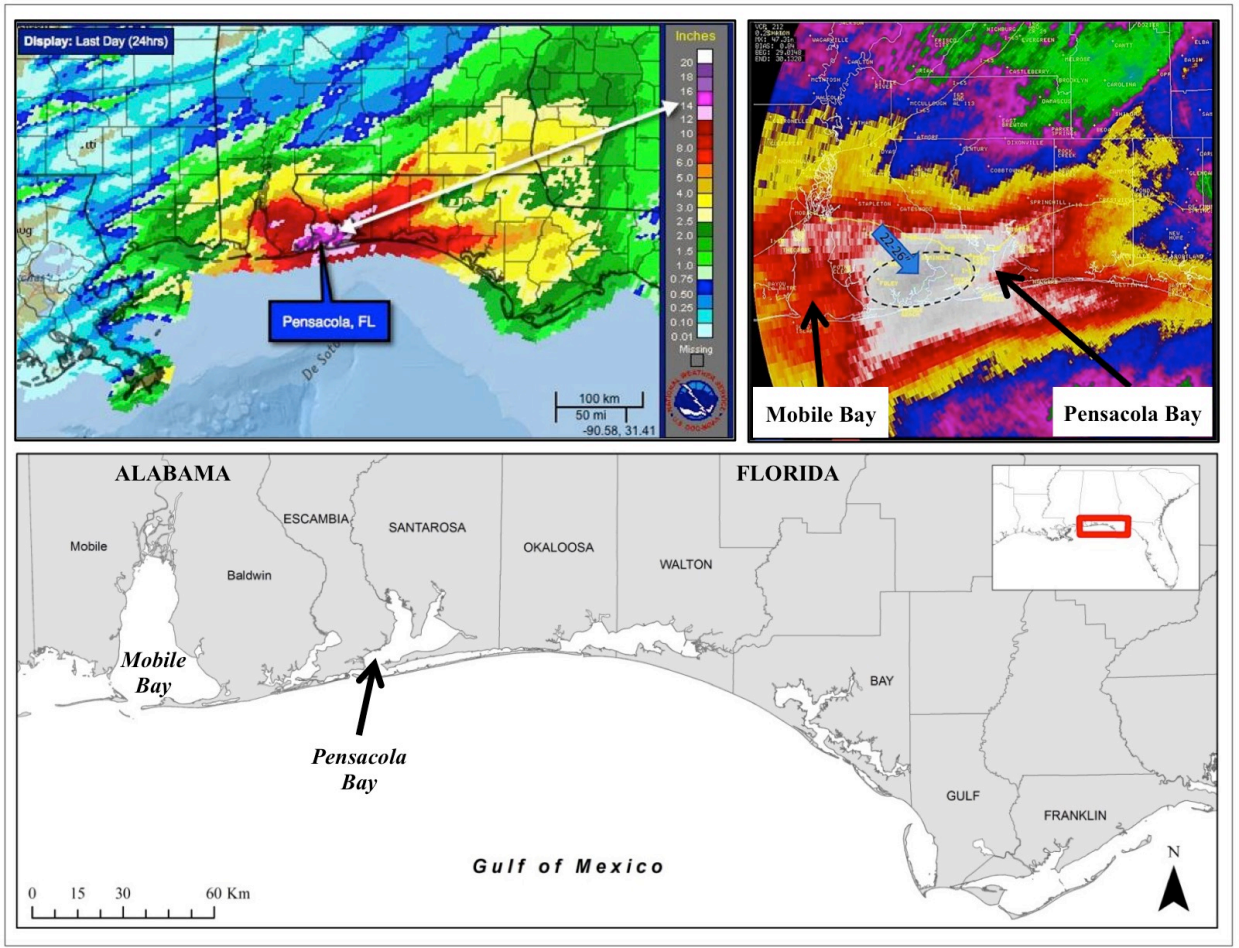
Geographic extent and radar from historic flood event, April 2014. Top left: estimated total rainfall over the two-day event, between 8:48pm on Monday April 28^th^ and 9:20am on Wednesday, April 30^th^; Top right: 24-hour radar estimated rainfall between 9am Tuesday, 29 April– 9am Wednesday, 30 April, zoomed in to show the most impacted region; Bottom: Stranding data coverage area and Pensacola Bay study site location; Figures obtained from NOAA National Weather Service, accessed on May 25, 2019: https://www.weather.gov/mob/2014_April29_FlashFlood.

**Fig 2:**
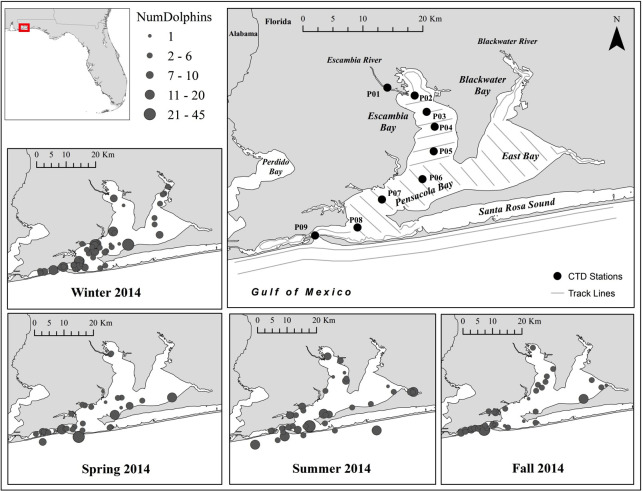
Study area showing survey area, track lines, and seasonal locations of all dolphin sightings (presented here as “best” estimates from the number of dolphins estimated in the field). Seasons defined in text; Coastal sightings were excluded for analyses; CTD stations are those monitored by the EPA (discussed in text) and are labeled in accordance with a previous publication [13].

On April 28–30, 2014 Pensacola, Florida, USA, experienced an historic, record-breaking flood event when it received over 20 inches of rain in 24 hours [Fig 1; 1, 2]. Studying the impacts of an unpredicted event is a challenge since answering questions about cause and effect requires comparing pre- event to post-event data, both of which are not often available. The effects of extreme flooding events on estuarine systems are poorly understood but can be hypothesized to have a substantial impact on the health of the ecosystem and its component organisms. The Pensacola Bay system may be particularly vulnerable given historic, widespread, water quality and sediment contamination issues [3–6]. The system receives heavy sedimentation from a vast watershed [3, 4] which, in combination with a history of industrialized land use, has resulted in a system with poor circulation and decreased natural flushing capabilities. Wastewater and storm water commonly carry high nutrient loads and contaminants, which then concentrate in local bayous where runoff drains. Groundwater contamination has been reported in association with local Environmental Protection Agency Superfund sites [7, 8]. Point and non-point sources of pollution have been shown to remain in the system at significantly contaminated levels over decades [6, 9, 10] and the Pensacola Bay system has been described as one of the most heavily polluted bays in Florida with respect to toxic substances [11]. Large rain events, such as the 2014 storm, are likely to exacerbate the problem by flushing unknown levels of fertilizer nutrients, bacteria, chemicals, and other land-sourced toxins into the bay [12], and sufficiently high winds can stir up contaminated sediments.

An additional complexity for understanding the impact of the 2014 storm, was the sheer volume of water that fell and flowed into the bay. The Pensacola Bay system is a relatively shallow estuary which covers about 370 km^2^ [3, 13]. While fresh surface waters are common from the watershed in the northern sections of the system [13], the extent of the freshwater influence resulting from this storm was unprecedented [2]. With a maximum elevation of only a little over 100 feet in Pensacola, standing water remained and drained for several weeks, likely leaving the bay system compromised for some time following the storm.

Within a week after the flood, common bottlenose dolphins (*Tursiops truncatus*) were observed in the Pensacola Bay system with skin lesions that had not been seen prior to the flood (i.e. winter 2013–2014). The relationships between skin lesion expression in delphinids and causal agents, or health implications, are poorly understood but have been linked to the presence of infectious pathogens [14–21] and changes in environmental conditions [16, 20, 22–29]. Evidence from reports around the Northern Gulf of Mexico (NGoM) have linked flooding, storm events and/or prolonged freshwater exposure to the presence of skin lesions in bottlenose dolphins [28–32]. Bottlenose dolphins residing in bays, sounds, and estuaries are known to be exposed to lower salinity waters due to runoff from watersheds and rivers (<15 ppt), but 8 ppt has been suggested to be a threshold for defining bottlenose dolphin habitat in at least one NGoM bay [Barataria Bay, Louisiana; 33]. Prolonged exposure to very low salinities (<5 ppt) is known to cause health concerns [25, 30–32] by adversely affecting blood serum chemistry and reducing immune system function, and can result in adrenal fatigue [23, 30–32, 34]. Furthermore, there’s a strong link between environmental stressors and the distribution of infectious pathogens [e.g. 35].

Given our observations of skin lesions on dolphins in the week following the flood, what was known at the time about the intensity of the event and contamination issues persistent in the Pensacola Bay estuary and known adverse health concerns that bottlenose dolphins face with prolonged freshwater exposure, it was hypothesized that dolphins inhabiting the inshore system would experience substantial health issues. Without the resources to capture dolphins for a proper health assessment [36], remote visual assessment of dolphins, specifically of skin lesions, is one of the only ways to provide insight on population health [37]. Measures of skin lesion prevalence (i.e. the proportion of animals in a population that have skin lesions) and extent of individual coverage (i.e. the proportion of epidermis covered in skin lesions for an individual) have been used to track lesion progression/regression over time and serve as indicators of population health [21, 22, 24, 38].

The timing of this flood was especially problematic because it occurred at the peak of the calving season (March and April). Within a week after the flood there were several neonate (infant) dolphin deaths in the Panhandle. If such events increase stress and related immune deficiencies [21, 27], increase mortality rates [25], and/or negatively impact reproduction rates and success [22, 39], then they have the capacity to adversely affect the entire population over time.

### Purpose

Here we assess the potential consequences of the flood on bottlenose dolphin health and mortality. Data from an ongoing population dynamics study were used to evaluate the relationship between skin lesions (prevalence, extent, and progression) and the flood (pre-flood conditions compared to one week, two months, and six months following the flood), with respect to changing environmental conditions (salinity). It was hypothesized that if skin lesions were linked to freshwater exposure, then lesion extent and prevalence would increase following the flood and decrease as the system returned to pre-flood conditions (i.e. as salinity increased in the bay again). Annual stranding records (2012–2016) from Alabama to the eastern Florida Panhandle were used as an indicator of dolphin health to test the hypothesis that the flood event resulted in increased annual mortality rates.

## Methods

### Ethics statement

Photo-ID data presented here were collected under a National Park Service permit number of GUIS-2015-SCI-0043 and the following NMFS Scientific Research Permits: 19062, 15543, and 20455. Data collection protocols were approved by Institutional Animal Care and Use Committee of the University of Central Florida (Protocol Number: 15-33W). Samples from stranded animals were collected under NOAA’s responsibility to the MMPA 1972 under Section 109(h), and a Stranding Agreement as part of the Marine Mammal Health and Stranding Response Act to respond to and collect samples from stranded marine mammals.

### Dolphin photo-identification

Bottlenose dolphin photo data for this project were utilized from an ongoing seasonal mark-recapture photo-identification project designed to assess baseline population dynamics for bottlenose dolphins that utilize the Pensacola Bay system [40] (30.41946, -87.09572) located in Pensacola, Florida, USA. Boat-based (6–7 m center console) field surveys (Fig 2) were conducted seasonally using Pollock’s robust design (with a schedule of 2–3 days per secondary session, followed by two mixing days, repeated three times, for a total of 10–13 field days per primary season) and recommended photo-ID protocols [41–44]. All surveys were conducted in sea conditions that did not exceed Beaufort 3. All fieldwork included trained personnel consisting of a boat captain and 2–3 observers. When dolphins were encountered, an attempt was made to photograph the left and right sides of the dorsal fins of all individuals present (with either a Nikon D200 or Canon 60D with 70–300 mm zoom lens). A dolphin group was considered as those within 100 m of each other, generally moving in the same direction and engaging in similar behavior [45, 46]. The following additional data were collected for every dolphin encounter: GPS location of dolphins, time, weather and sighting conditions, dolphin group size and behavior, and an estimated number of each age class (i.e. adults, calves and neonates). Calves were defined as being approximately 50%-75% of adult length and swimming in infant position [47]. Neonates were distinguishable by the presence of fetal folds (approximately 5–7 light vertical stripes), darker coloration, uncoordinated surfacing patterns, and with body lengths less than half that of the adult [47, 48]. At the beginning of each sighting, temperature, salinity, and dissolved oxygen readings were taken of surface waters using a ©YSI instrument (Model 85). Photos were graded based on fin distinctiveness and photo quality and individuals were cataloged according to established methods [43, 44, 49–52] using a Microsoft Access-based database program, FinBase [53]. Dolphin sightings from along the open Gulf coast were excluded since inshore dolphins were more likely to have been exposed to the flood and as such, were the focus of this project.

### Skin lesions

Photos were evaluated for purposes of characterizing and quantifying skin lesion prevalence and extent before and after the flood event using four seasons of data. “Pre-flood” survey data included winter 2013–2014 (December 30, 2013, to January 14, 2014). “Post-flood” survey data included the following three seasons: spring 2014, which began the week immediately following the flood and included May 06–19, 2014; summer 2014, two months following the flood (July 01–13, 2014); and fall 2014, about six months following the flood (October 10—December 14, 2014). The time periods included were based on data available from surveys already planned and funds already secured for the population dynamics project. The fall 2014 dataset extended over a longer time period than the other included seasonal datasets and only included two secondary sessions instead of three (i.e., each section of the system was only covered twice instead of three time). This was due to inclement weather that year (preventing us from being in the field consistently) and insufficient funding at the start of that field effort. Four surveys were completed in October and two were delayed until the first week of December. Consequences of this variability are discussed below.

Given the subjectivity in quantifying skin lesions from photographs and challenges realized due to rater uncertainty in identifying skin lesions, the photo screening process described in Toms et al. [37] was updated and re-evaluated for inter-rater reliability (elaborated on in S1 File). After cataloging photographs, unique individuals identified in each season were queried. All unprocessed photos for each unique individual were pulled, organized by season, and visually examined and scored for skin lesion presence/absence (on any area of visible body, regardless of photo quality) and rater certainty of presence. Presence was determined using examples and definitions outlined in Toms et al. [37]. Rater certainty was assessed using a categorical rating of low (unsure if lesion is present), moderate (likely lesion present but not 100% certain), or high (very confident of lesion presence), and was impacted by number of photos available, overall PQ, amount of body visible, lighting, and sharpness of focus on the area of body in question. Physical deformities, rake marks, scars and other injuries were not included. An animal was removed from inclusion of any dataset if the photos were not of good enough quality to make an assessment about presence/absence. If the animal was coded as “no skin lesions present”, then the rater needed to have a certainty of moderate or higher, otherwise, there was clearly not enough information to make this assessment and the individual was excluded from all datasets.

If an animal was assessed to have skin lesions present, then it had to pass at least one of the following minimum conditions to be included in the dataset: (1) certainty in skin lesion presence was high and there was at least one photo showing the lesion that was in excellent focus (where excellent focus = 2, moderate = 4, and poor = 9) [52], (2) certainty was high but if no photo showing the lesion had excellent focus, then two or more skin lesion photos were required and at least one of these must have moderate focus, (3) if certainty was moderate, then at least two photos showing the lesion were required and both had to have a photo focus score of moderate or better (≥ 4). If the scored confidence for skin lesion presence was low, the dolphin was included in the prevalence calculation as “without lesions” (since the alternative of excluding all low confident scored individuals for prevalence would have resulted in artificially inflating the final estimates). Lesion types were categorized for descriptive purposes, based on a simplified version of the classification system evaluated in Toms et al. [37]. Given the challenges previously described from visually distinguishing between a large number of categories [37], the original 17 categories were collapsed into six: (1) potentially pathogenic (PP); (2) rake mark-associated potentially pathogenic (RMA–PP); (3) orange; (4) hypopigmentation (included any light non-PP patches of unknown etiology); (5) hyperpigmentation (included all non-circular, non-PP, dark patches that were of unknown etiology); (6) discolored head and/or nuchal patch. PP lesion types that occurred on fresh tooth rake marks were distinguished from those not on rake marks and given a separate certainty rating (further detailed in S1 File). All lesion types were recorded for all individuals, even if they presented with more than one type.

The prevalence of skin lesions was estimated as the proportion of individuals in the population (for which photos were of good enough quality to score lesion presence/absence) with at least one of any type of skin lesion that passed the lesion screening process above. If an individual was seen more than once in a given season, skin lesions were only recorded once as “present”.

**Fig 3:**
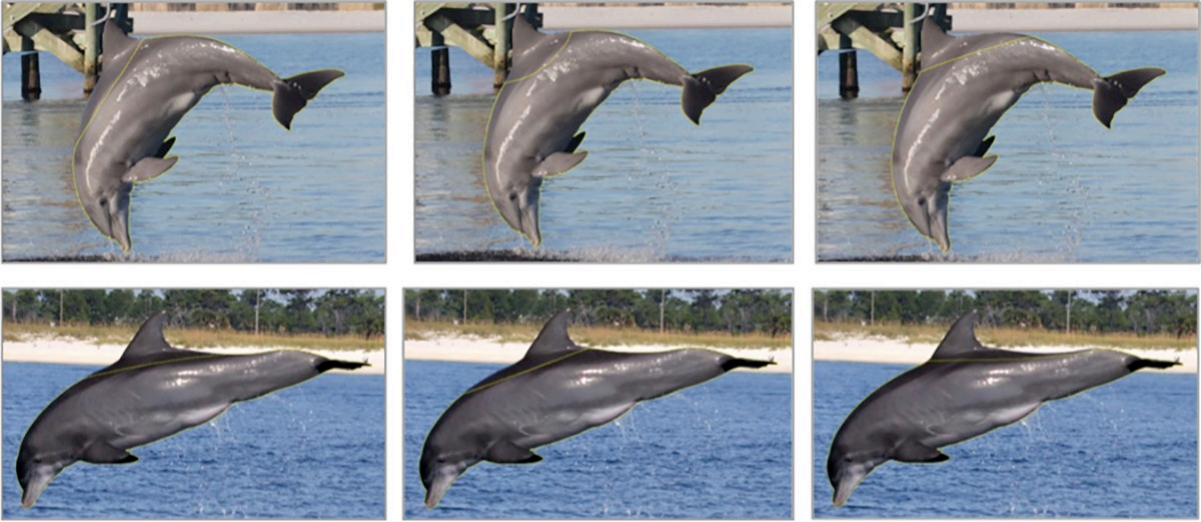
Traces of dolphin bodies used to determine what 10% of the dorsal surface would look like at different surfacing angles. Traces completed in Image J by calculating the number of pixels traced of the entire dolphin and then excluding 10% of those pixels from the dorsal surface.

In order to be included for measures of extent, individuals needed at least one picture that met photo quality standards above and was showing ≥ 10% of the body above the surface of the water [as implemented by 39]. A visual aid was created to determine this cutoff using program Image J to trace the body of a dolphin leaping out the water in a photograph (Fig 3). Lesion extent was measured two ways: using a categorical rating approach and by digitally tracing photos [as in 37]. The categorical approach allowed us to include lesions that were impossible to trace (i.e. those with poorly defined edges such as orange hue or mottling along the entire flank of an animal) and include cases where tracing accuracy may have been impacted by the angle of the dolphin to camera. The estimates from tracing data were alternatively expected to offer a more precise measurement and a resulting continuous variable, which can potentially offer higher analytical power over categorical variables. Given these differences, we expected that both measures would be important for interpretation. However, when results from the two measurements methods were compared (discussed in S1 File), categorical ratings were overall more likely to be accurate and captured 30%-40% more of the data available to measure, compared to estimates using tracing methods. Therefore, here we only discuss data and results using the rating approach. Details on the methodological comparisons are presented in S1 File as a guide for future studies.

Lesion extent ratings were scored for the visible portion of the animal as follows: background levels (<5% coverage of visible epidermis), low (5–20% coverage of visible epidermis), moderate (20–50% coverage of visible epidermis), and high (>50% coverage of visible epidermis). This division followed Bearzi et al. [39] with the exception that the lowest category was split into two. We felt the “background” category would be more appropriate when comparing changes over time in a system where background levels may be present prior to the storm, given the known environmental issues in the area. Overall extent of a given individual was scored after first assessing the left and right side independently (when available). In cases where only one side of the animal was available, the overall score was the same as the single side available. A visual reference was used when scoring extent categories to improve accuracy. Other photo examples of skin lesions were traced to create a set of reference images showing the true percentages of visible skin lesions ranging from 0.13% to 74% coverage (and included examples for both dorsal fin only and dorsal fin and body combined).

McNemar’s exact tests for analyzing repeated measures with dichotomous variables were used to evaluate changes in prevalence and extent of lesions over time. Data included only those individuals seen prior to the flood that were again seen at least once following the flood (spring, summer, or fall, 2014). Individuals only seen once were excluded from these analyses. Given that some individuals were seen in multiple seasons but not every season, prevalence analyses were conducted two different ways. First, the presence of skin lesions following the flood was scored for any individual seen with skin lesions at least once in any post-flood season (spring, summer, and fall 2014). Pre-flood data (winter 2013–2014) were compared to this collapsed single, post-flood variable. Second, given biases associated with collapsing data across seasons, paired tests between every post-flood season compared with the pre-flood season were conducted separately (with a Bonferroni adjustment of significance level for multiple tests).

Because very few dolphins demonstrated skin lesion extent greater than 20% of the exposed skin (regardless of measure used) extent results were turned into a dichotomous variable for those individuals exhibiting <5% lesion extent (corresponding to a rating score of 0 or 1) and those exhibiting ≥5% lesion extent (rating score ≥2). The McNemar’s test was used to determine if the proportion of individuals with skin lesion extent ≥5% increased from pre-flood to post-flood (collapsed across spring, summer, and fall seasons). For any individual seen in more than one post-flood season, measures were used from the season with the most progressive skin lesions. Season-by-season paired comparisons of extent were not analyzed due to too many zeros for contingency table-based analyses.

### Stranding reports

Marine Mammal Stranding Networks across the NGoM respond to cetacean strandings. A standard set of demographic data (called Level A data) are collected for stranded marine mammals and submitted to the Marine Mammal Health and Stranding Response Program (MMHSRP) National Stranding Database. Stranding reports included: species, date, stranding location (including state, county, latitude and longitude, if known), carcass condition, sex, length, and date of initial observation. Level A data were obtained from the MMHSRP for all events reported from Alabama through the Florida Panhandle, to Franklin County from 2012–2016 (raw count data presented in S2.1 Table in S2 File). Alabama was included since the storm came from the west and swept across Mobile Bay (connected to Pensacola Bay inshore through the Intracoastal Waterway), causing massive flooding in Alabama as well [e.g. Fish River in Baldwin County, Alabama, rose to historic levels; 2]. Data from a larger region was also more likely to be meaningful in the context of the storm since surface currents can carry carcasses to shorelines away from where the animal died [54, 55]. Data through to Franklin County, Florida were included as a direct comparison to recent publications that evaluated stranding data [56, 57], overlapping in time (2010–2013) and spatial scale with the data evaluated here (S2.1 Fig in S2 File). The timing of the flood overlapped with an ongoing Unusual Mortality Event (UME) in the NGoM from 2010–2014 [UMEs are government-declared events, identified by a working group of experts using multiple criteria based on their dissimilarity compared to the normal stranding patterns; 58]. The Florida Panhandle (ranging from the Alabama-Florida border through to Franklin County, FL) was deemed the eastern geographic extent of the UME [56], providing a useful dataset for comparison. Eastern counties in this range were not impacted by the flood and therefore also provided a non-flood-impacted reference group for comparisons.

Data were organized by date and location and used to calculate the total number of cetaceans stranded, total numbers of *T*. *truncatus* (herein referred to as *Tt*) stranded, and total *Tt* perinate strandings (from final stages of pregnancy and/or immediately following birth). Perinates were defined as those calves less than 115 cm in length (rostrum tip to fluke notch) based on previous reports of average lengths at birth [59, 60]. Data in which the carcasses were reported as “partial” were eliminated from these counts. Number of dead stranded animals was also calculated where dead was defined as any individual that stranded dead, died after stranding, or was euthanized over the time of the report. Animals that were last seen alive, in unknown condition, or with an unknown fate were coded as “alive”. Data for all cetaceans were included for summary purposes but analyses were limited to *Tt* since this species comprised of ~80% of the stranding data for this time period and it is the only species found inshore, and thus the only one likely to be directly impacted by the flood.

In order to examine changes in stranding rates over time, in response to the flood, data were organized by year where “pre-flood” years were 2012–2013, “flood year” was 2014, and “post-flood” years were 2015–2016. Data from each county from Alabama to the Eastern Florida Panhandle were then grouped based on flood-impacted zone. Rainfall reports were examined by county for April 28–30, 2014, using the CoCoRaHS public database and weather reports [2; https://www.cocorahs.org/]. The following coastal counties reported daily precipitation values of 10 inches/day, or more, across rainfall monitoring stations (listed west to east) and were considered to be “flood-impacted’: Mobile (AL), Baldwin (AL), Escambia (FL), Santa Rosa (FL), and Okaloosa (FL) counties (Fig 1). Non-flood-impacted counties included the remainder of the coastal Florida Panhandle counties that were not directly hit by the storm and for which rainfall values were less than 10 inches per day for the two-day event. These counties included Bay, Gulf, and Franklin counties and collectively provided a control group. If the flood had a measurable effect on stranding rates, then detectable increases should be limited to the impacted region, otherwise non-flood related factors were likely also involved. The “flood-impacted” group was further delineated by Alabama and Florida counties since pre-flood (2012–2013) conditions were expected to differ regionally. Alabama was still impacted by the ongoing UME leading up to the flood [57] given that stranding rates in 2013 remained elevated compared to a historic baseline for the region (i.e. confounding the “pre-flood” dataset for Alabama in this study). Conversely, although the Florida Panhandle was included in the original geographic extent of the UME, monthly stranding events for this region were not elevated above historic baseline during any period following the start of the UME, except for in June and July of 2012.

Stranding count data were analyzed using a negative binomial log-linear regression model, which included region (Impacted-Alabama; Impacted-Florida; non-impacted-Florida), year, and a region x year interaction term. The linear model was used to test whether strandings increased in response to the storm and remained elevated. The negative binomial log-linear regression model used in analyses was chosen because of evidence for overdispersion of the count data (*φ* = 2.13). This model demonstrated reduced residual deviance compared to the alternative of correcting for standard errors using a quasi-Poisson GLM model (data not presented here), and the final model accounted for most of the overdispersion (final *φ* = 1.32). A GLM equivalence of R^2^ (i.e. explained deviance) was calculated with the result of 6.5 [calculated as 100 * (null deviance—residual deviance) / null deviance; 61]. The negative binomial model selected was compared using log likelihood to the same model using a Poisson distribution. The negative binomial distribution was a significantly better fit (*d* = 67.07 (4); *p* < 0.001). Non-significant terms were dropped, and the model was updated until only terms that significantly contributed to the model remained. Model selection was completed using both an analysis of deviance (a hypothesis testing approach using a Chi-squared test) and a stepwise AIC model selection tool. Models were also examined separately using a quadratic term (i.e. tested for a non-linear relationship) to test whether 2014 showed a significant peak in stranding events that then dropped in subsequent years.

### Environmental data

To better understand the significance of the pulse of freshwater entering the Pensacola Bay system and whether or not dolphins would have been exposed to unusual quantities of freshwater for long periods of time, several sources of environmental data were examined. The Environmental Protection Agency (EPA) has monitored a variety of water quality parameters using a CTD (instrument designed to measure conductivity, temperature, and depth, among many other parameters) at nine stations spaced at ~3 km intervals along a longitudinal transect of the Bay from the Escambia River to the NGoM at Pensacola Pass (Fig 2). The transect data provide a profile of water quality in the system for a time period that includes this project, enabling us to generate plots similar to that presented in Hagy and Murrell [Fig 5 in 13]. Plots enabled us to visualize in 2-dimensions the effect of the intensity and duration of freshwater inputs on water temperature and salinity in the months following the flood and compare these patterns to previous summaries of freshwater flow during “normal” conditions [13, 62]. Water quality data were collected in 0.25 m vertical bins at each station spanning the full depth of each station (2–11 m) Data were available for the following dates in 2014: 04/21/2014, 05/13/2014, 06/04/2014, 06/24/2014, 07/23/2014, 08/20/2014, 09/17/2014, 10/15/2014, 11/12/2014, and 12/10/2014. Another indicator of freshwater input in the system was obtained from data collected at a USGS-maintained flow gauging station near Molino, FL [site 02376033; 62]. This site is the principal source of freshwater to Escambia Bay and 60% of the watershed to the Pensacola Bay system flows through it [watershed drainage area of 10741 km^2^: 13]. Data on daily precipitation and mean daily discharge measurements were downloaded from the USGS website (https://waterdata.usgs.gov/usa/nwis/uv?site_no=02376033) for all time periods available (which were limited to: June 1, 2013—December 31, 2015) in the three years surrounding the flood. To evaluate changes in discharge rates and average precipitation in the years before, during, and after the flood, a Kruskal-Wallis test was performed on natural log transformed data.

## Results

### Environmental data

**Fig 4:**
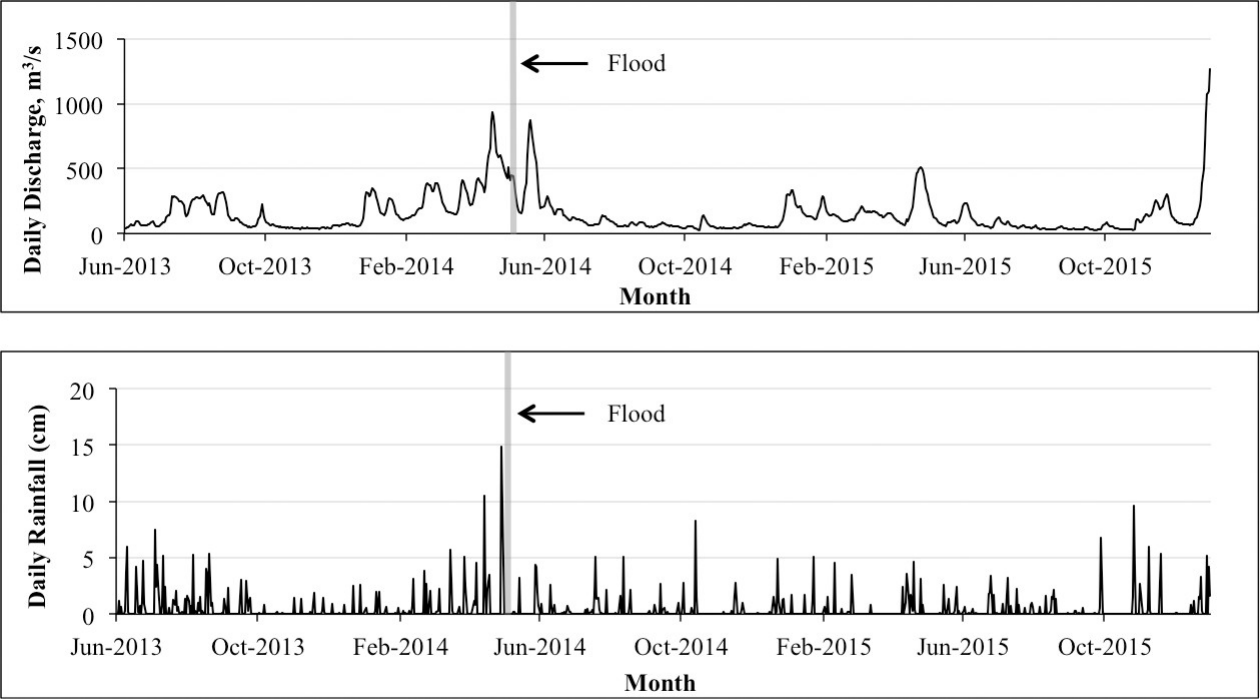
Daily rainfall and discharge data surrounding the flood event. Above: daily freshwater discharge from gauging flow station; Below: daily rainfall measurements; Data obtained from USGS Molino, FL flow gauging station (02376033) near the mouth of the Escambia River.

Rainfall data demonstrated that rainfall on the day of the flood easily surpassed that of any other rain event in a three-year period and that another significant rainfall event proceeded it, resulting in discharge well above average (Fig 4). For the three-year period analyzed (noting that data for Jan-May were not available for the 2013 estimates), monthly average discharge through the Escambia River station varied from 37 to 541 m^3^/s (i.e. 0.29–4.35 mm/d for the Escambia River watershed area), and monthly average precipitation varied from 0.29 to15 mm/day (0.03–1.5 cm/day). The monthly max for each of these occurred in April 2014 and surpassed “high flow” conditions previously characterized for this system (data from 2002–2004), whereby monthly average precipitation ranged from 1 to 11 mm, and monthly average discharge for the Escambia River was 431 m^3^/s [13]. While certainly a significant event, this pulse flood did not result in statistically significant increases in annual estimates of average discharge (*H* (2) = 1.088, *p* = 0.58) or average rainfall (*H* (2) = 0.88, *p* = 0.88) for 2014 compared to 2013 or 2015.

**Fig 5:**
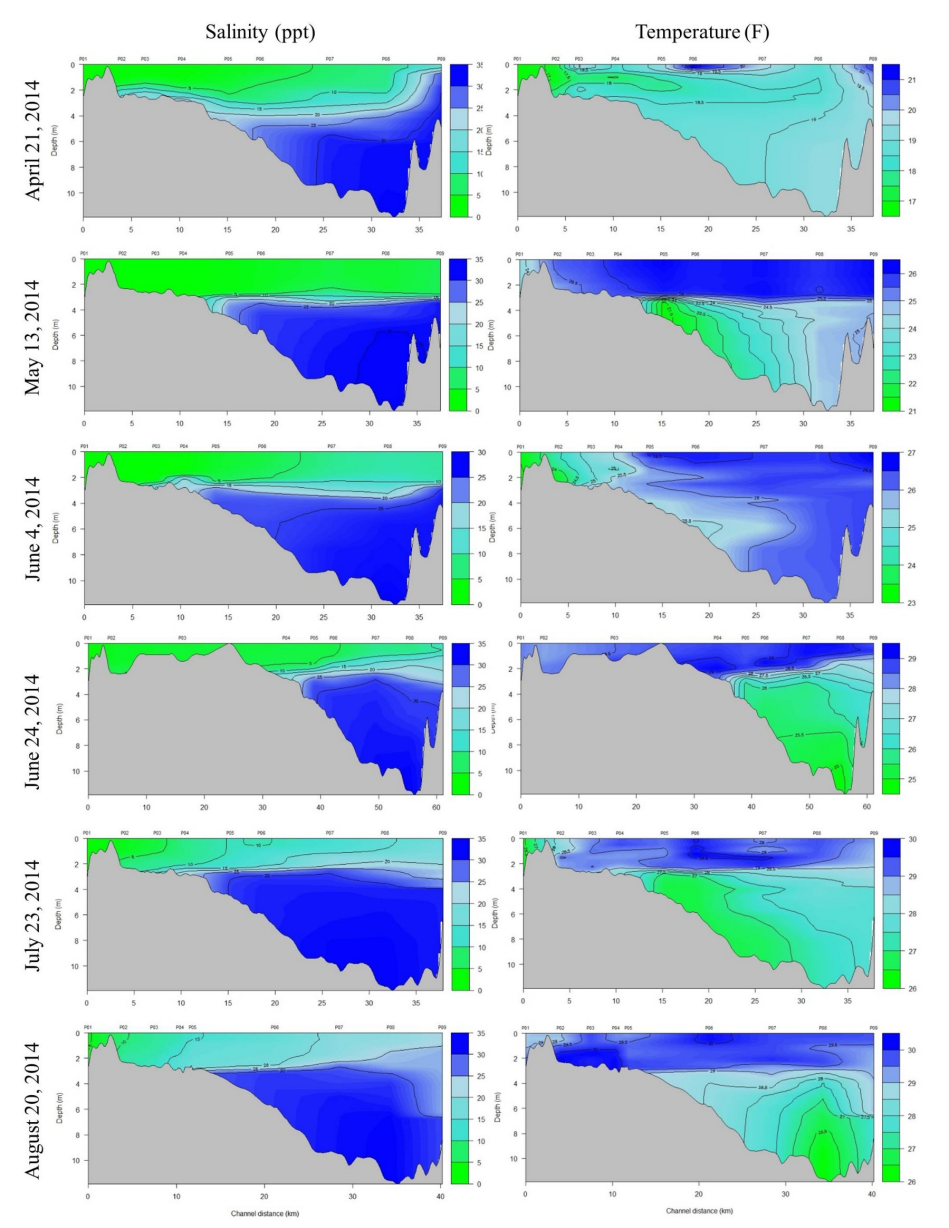
Salinity-depth profiles from EPA CTD stations along a transect that runs through the Escambia-Pensacola Bay system (see Fig 2) for April–August 2014. Station numbers are listed across the top (P01-P09) where P01 is located at the mouth of the Escambia River and P09 is at the mouth of Pensacola Pass. Escambia Bay opens into Pensacola Bay roughly at station P05; Channel distance is the distance from station P01.

**Fig 6:**
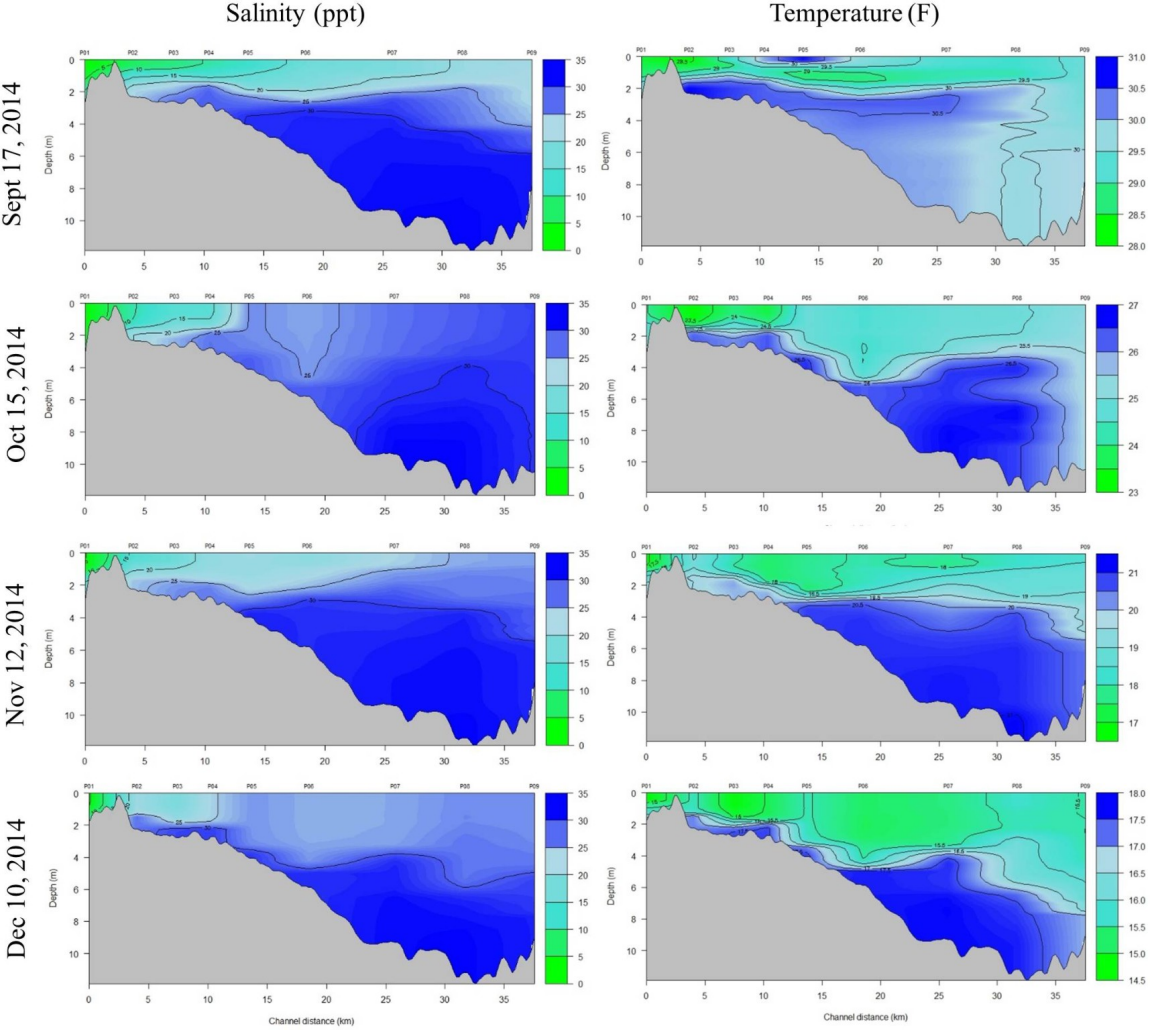
Salinity-depth profiles from EPA CTD stations along a transect that runs through the Escambia-Pensacola Bay system (see Fig 2) for September—December 2014. Station numbers are listed across the top (P01-P09) where P01 is located at the mouth of the Escambia River and P09 is at the mouth of Pensacola Pass. Escambia Bay opens into Pensacola Bay roughly at station P05; Channel distance is the distance from station P01.

The extensive freshwater influx from the flood is readily apparent by the CDT plots in Figs 5 and 6; measurements taken about two weeks after the flood (May 13, 2014) show a completely fresh, warm (~26°C) water surface lens of 3–4 m deep that extended all the way to the pass where the bay meets the NGoM. Although somewhat reduced, this lens was still evident a month later (June 4^th^ and 24^th^). Surface salinities didn’t start resembling what would be expected for the bay during the summer months [13] until July, although most of Escambia Bay was still experiencing salinities <5 ppt until August.

### Skin lesions

In total, 333 unique individuals were seen across the four seasons analyzed (sample sizes by season are presented in Table 1). 187 of these were only seen once during this period. 146 were seen more than once across the four seasons. 67 (20%) unique individuals were seen with skin lesions (after screening for photo quality) at some point but 24 of these were only seen in one of the four seasons.

**Table 1:** Numbers of unique dolphins seen each season and the numbers of individuals utilized to estimate skin lesion prevalence and extent.

Cluster	Total unique dolphins	Total available for scoring ^a^	No. dolphins IDed with skin lesions before screening ^b^	No. retained to estimate prevalence	No. retained to estimate extent ^c^
Winter ‘13-‘14	164	135	18	13	13
Spring 2014	108	94	33	25	25
Summer 2014	137	103	35	18	15
Fall 2014	127	105	39	30	29

*Note*:

^a^ Refers to the number of individuals for which there were good enough photos to determine presence/absence of lesions

^b^ Counted if they were scored with moderate or higher level of certainty but before applying photo quality and/or spotted lesion criteria for inclusion

^c^ Extent was not scored for any individual that did not have photos showing ≥10% of the body

See text and S1 File for additional details on these criteria.

Lesions were seen on dolphins across all seasons, even prior to the flood (although in low frequencies; Table 2). Prevalence estimates were elevated immediately following the flood (spring 2014: 27% of unique individuals had lesions) compared to the winter prior (9.7%) and remained elevated through the end of the year (18% and 29% for summer and fall, respectively). The most common type of skin lesion seen in every season was potentially pathogenic (PP). Lesions in this category were seen both in association with rake marks and on dolphins without any rake marks. Prevalence of hypopigmentation and PP lesions on non-raked skin doubled from winter to the spring after the flood and stayed elevated over time. One might expect darker lesions to turn to lighter patches as they heal over time and therefore the ratio of hyperpigmentation to hypopigmentation to change accordingly. This was not the case here as there were consistently more hypopigmentation cases and very few cases of hyperpigmentation; however, several darker lesion types like tattoo lesions and dark, black spots were included in the PP category instead, so hyperpigmentation only included remaining dark patches of unknown etiology, not all hyperpigmentation cases.

**Table 2:** Prevalence (with sample size in parentheses) of skin lesions seen on dolphins found in the Pensacola Bay system over time and by category.

	Total Dolphins ^a^	Total Prevalence	PP	RMA-PP only	Orange	Hypo-pigmentation	Hyper-pigmentation	Discolored Head and/or Nuchal Patch
Winter ‘13-‘14	135	0.097 (13)	0.067 (9)	0.030 (4)	0.007 (1)	0.024 (4)	0.007 (1)	0.007 (1)
Spring 2014	94	0.266 (25)	0.191 (18)	0.096 (9)	0.043 (4)	0.096 (9)	0.053 (5)	0.021 (2)
Summer 2014	103	0.175 (18)	0.126 (13)	0.049 (5)	0.029 (3)	0.087 (9)	0	0
Fall 2014	105	0.286 (30)	0.181 (19)	0.067 (7)	0.114 (12)	0.048 (5)	0.067 (7)	0.029 (3)

*Note*:

^a^ Prevalence calculated based on the total number of individuals that had photos good enough for scoring presence/absence of skin lesions

Positive scores were excluded if certainty of lesion presence was low; Individuals may have had more than one skin lesion type counted; The flood occurred one week prior to the spring, 2014 season; PP: potentially pathogenic; RMA-PP only: rake mark-associated (RMA)–potentially pathogenic (PP).

71 dolphins seen during the winter prior to the storm were resighted one or more times in the three seasons following the flood. Sixteen of these that did not show lesions prior to the flood (winter 2013–2014), were seen with lesions at least once following the flood, resulting in a statistically significant increase in the proportion of individuals with skin lesions from pre- to post-flood (data collapsed across post-flood seasons; McNemar’s exact test; *n* = 71; *p* < 0.001). When each post-flood season was compared independently to the pre-flood season however, not all demonstrated a significant increase in prevalence. Of the 33 individuals seen in both the winter and spring, there was no significant change in the prevalence of skin lesions over that time period. The same was true for the comparison of the 33 individuals seen in both winter and summer (McNemar’s exact tests with Bonferroni adjustment for multiple tests; *p* > 0.02). However, there was a significant increase in the proportion of dolphins with skin lesions in the fall compared to those same individuals (*n* = 38) that were seen prior to the flood (*p* = 0.01).

Estimates for lesion extent are summarized in Table 3 by season and estimate type. The most common lesion extent category scored across seasons was for the trace/background levels category (i.e. individuals with <5% of the skin covered by skin lesions). There were only 11 scores of lesion extent greater than 20% and these were scored in spring (immediately following the flood) and fall but not during the summer season. Only one individual was given a score of “high” extent (>50% coverage) in any season (across all measures) and it was only seen in the spring, immediately after the flood [40]. There was a significant increase in the proportion of dolphins seen post-flood with lesion extent ≥5% compared to pre-flood (McNemar’s exact tests: *n* = 68, *p* = 0.001). Of the 67 unique individuals seen with lesions at least once across the four seasons, 33 of them were seen with an extent ≥5% at least once across the four seasons and 21 of these were seen more than once across the time period analyzed. A visual representation of the changes in extent of these 21 individuals is presented in Table 4.

**Table 3:** Extent of skin lesion coverage measured as categorical ratings.

	Lesion Extent Rating				
	1	2	3	4	Total
Winter ‘13-‘14	12	1	0	0	13
Spring 2014	16	5	3	1	25
Summer 2014	10	5	0	0	15
Fall 2014	10	12	7	0	29

*Note*: Lesion rating categories: 1 = background levels (<5% coverage of visible epidermis); 2 = low (5–20% coverage of visible epidermis); 3 = moderate (20–50% coverage of visible epidermis); 4 = high (>50% coverage of visible epidermis); ≥10% of the body had to be visible in photos in order to be scored for extent.

**Table 4:** Lesion extent progression over time in 21 individuals that demonstrated lesion extent ≥5% at least once in the four seasons evaluated.

ID	Winter ’13-’14	**Flood**	Spring 2014	Summer 2014	Fall 2014
374				2	0
376	0				2
426	1		2		
465	1			0	2
483			2		0
511			0		2
513	0		0	0	3
537	0		3	2	
559	1		0		2
629	2		1	0	3
634			0		3
645			2	2	1
650	0		3		3
657	0		3		
663			2	1	1
670^^^	1^^^		1		2
671*	1*		1	0	0
677	1		0		2
682*	0		1*		
699	0		0		2
933	nd			2	
blank	not seen				
0	No visible lesion				
1	Trace levels of extent (<5%)				
2	Low Extent (5–20%)				
3	Medium Extent (20–50%)				
No high extent cases					

*Note*:

^Photos available during the winter 2013–2014 season for ID 670 were not good enough quality for inclusion in analyses, however, certainty of skin lesion presence was high and therefore included in this descriptive presentation

*Dorsal extent was traced as >5% but the rating results were < 5% and the dorsal fin + body trace was <5% for ID 671 and unavailable to trace for ID 682; nd: seen and photos taken but not of good enough quality to determine skin lesion presence.

### Stranding records

**Fig 7:**
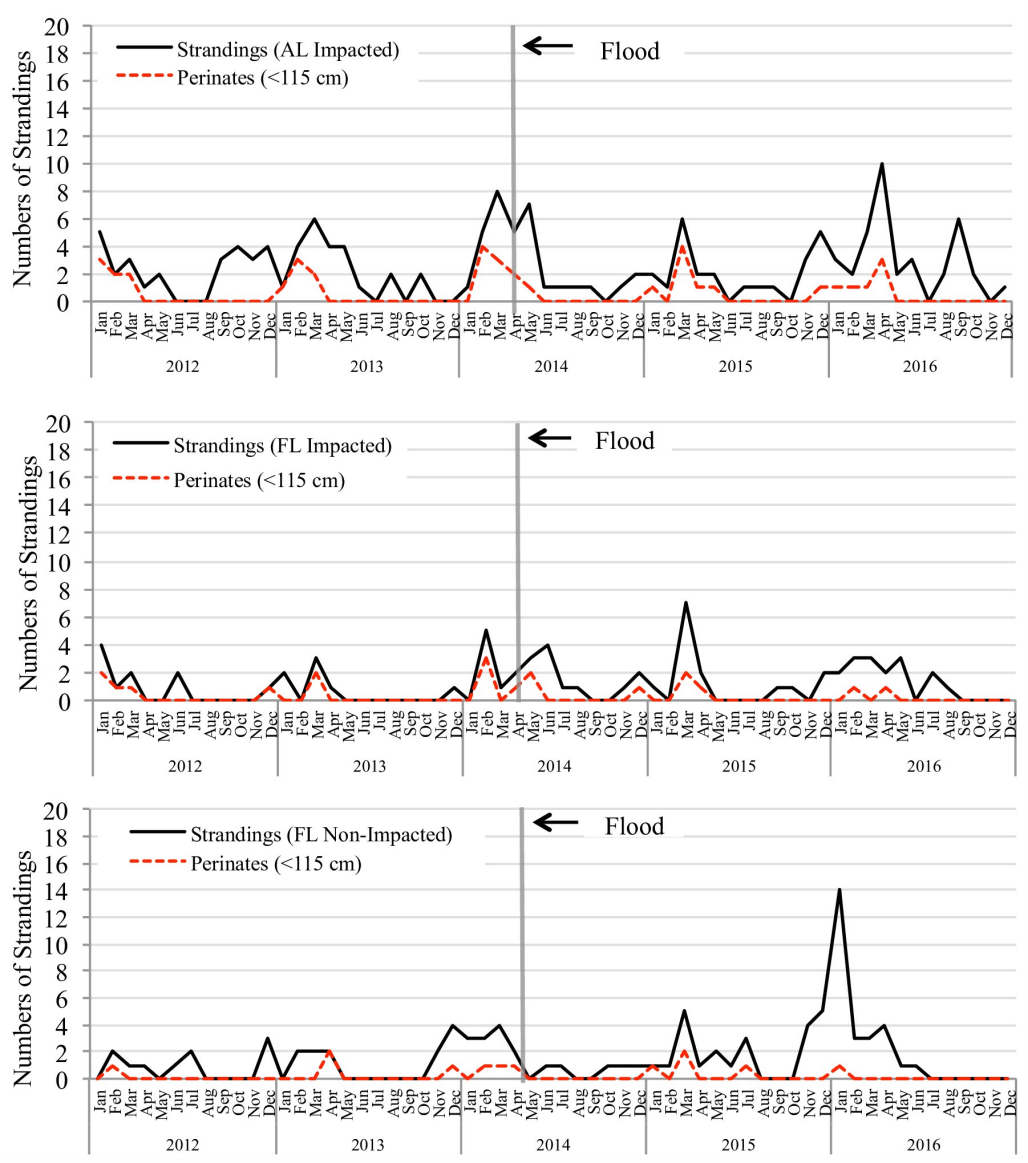
Total number of bottlenose dolphins (*Tt*) and *Tt* perinates stranded per month and year, per region, from 2012–2016. Top: Data from Alabama counties impacted by the flood (Baldwin and Mobile counties); Middle: data from the Western Florida Panhandle counties impacted by the flood (Escambia, Santa Rosa, and Okaloosa counties); Bottom: data from the Eastern Florida Panhandle counties that were not impacted by the flood (Walton, Bay, Gulf, and Franklin counties).

Stranding data by region, summarized across years, are presented in Fig 7. The full regression model for analyzing changes in stranding events across time was not significant. Both model selection approaches (analysis of deviance and stepwise AIC selection) determined that ‘year’ and the interaction term did not contribute significantly to the model, leaving ‘region’ as the only significant term (deviance = 15.4 (2), *p* < 0.001). Residual plots in the final model were examined and did not reveal any concerning patterns. Results from the regression model show that the numbers of strandings in Alabama were significantly greater (~64% higher, *IRR* = 1.64, *CI*: 1.05–2.56, *p* = 0.030) than for the reference group (Florida non-flood-impacted region), but strandings in the Florida flood-impacted region were not. A post-hoc analysis was conducted using a Tukey’s *p*-value adjustment for a three-way comparison (and the Sidak method for adjusting confidence intervals for multiple comparisons). Results demonstrated that the numbers of strandings reported in Alabama were also significantly greater than the flood-impacted region in Florida (*HSD* = 2.40, *p* = 0.001, *CI*: 1.72–3.35). Models fit with a quadratic term for year did not differ from the linear model.

## Discussion

The rainfall volume associated with the 2014 flood was so extraordinary that it has been described as a historic, 1 in 100 year or 1 in 200-year event [depending on the location where rainfall was estimated; 2]. Both the volume of water and the speed at which it was dumped into the area were unprecedented. There was another storm on April 14^th^ [63], 14 days prior to the flood. While substantial rainfall was recorded on April 14^th^ [63; also evident from large freshwater volume seen right before the flood, in the CTD plot on April 21^st^] and resulted in a spike in watershed drainage into the system, the volume from that storm paled in comparison to the flood event. Assuming the event had 25 inches of rainfall in 24 hours then direct rainfall replaced about 21% of the bay volume. Taking into account what is known about flow rates into the system from the local watershed [13], and with the assumption that runoff was equal to the rainfall given that the rainfall on April 14^th^ likely saturated the ground before the flood started, the total freshwater input into the system was roughly equal to three times the bay’s volume from this single flood event (see calculations in S3 File). Interestingly, daily discharge rates associated with the flood were lower than rates for the smaller storm right before it (April 14^th^). This was likely due to the ground already being saturated from the previous rain event, in which case discharge was already elevated and runoff from the flood was likely rapid. There were no significant increases in discharge rates into the system or rainfall when analyzed on an annual scale, indicating that even with both storm events in one year, these were pulse events as opposed to a part of an unusually high rainy season in 2014.

CTD stations were not in place for the East Bay section of the system to determine if the freshwater lens there was as strong or persistent as elsewhere in the bay system. However, past work in this system monitored a similar transect of CTD stations from Blackwater Bay through East Bay, intersecting the main system at CTD station P06 (Fig 2). Trends of low, moderate, and high flow rates described for the Escambia–Pensacola transect were mirrored by the East Bay–Pensacola transect [13] and under high flow rates, the upper half of the East Bay system was fresh from top to bottom. Therefore, it’s likely that the entire Blackwater and East Bay system was also fresh after the 2014 flood.

### Stranding data

Interpreting the level of impact on the health of the local dolphin population is challenging. While there was no significant peak in stranding events in 2014, as might have been expected, the stranding records showed that raw counts (S2.1 Table in S2 File) for all cetaceans increased ~61% from 2013 to 2014 and for the Florida Panhandle alone, nearly doubled (from 26 to 47 stranded cetaceans in 2013 and 2014, respectively), which was substantially higher than the historical annual average of 21 for this region [56]. A large proportion of stranded cetaceans recovered annually were bottlenose dolphins (*Tt*, 75%-85% annually and 80% overall). When the bottlenose dolphin data were subdivided by region, counts also nearly doubled from 2013–2014 (including those for just perinates) and stayed elevated in subsequent years, in the Florida-impacted region and in Alabama (although note that the increase from 2013 to 2014 for the Alabama data is not as dramatic).

Stranding events are challenging to interpret since many animals that die are either never recovered or are carried by currents to shorelines away from where the animal died [54, 55]. Even under nearly ideal circumstances, in a smaller estuary (Sarasota Bay) with high levels of human activity, only one third of bottlenose dolphin losses are actually recovered as carcasses [64]. Therefore, the numbers of animals that die during any time period are likely higher than measured and a lack of statistical significance doesn’t necessarily equate to lack of significance to the population(s) that were impacted. The power to detect significant difference across time may also have been masked by two other factors. First, as mentioned earlier, an UME was declared in 2010 in association with the *Deepwater Horizon* oil spill. This lasted through 2014 and became one of the largest and longest cetacean UMEs on record in the NGoM [56]. Reports from this UME concluded that the Florida Panhandle did not experience stranding events at elevated levels compared to those waters that were more directly hit by the oil spill to the west [56, 57], but Alabama did; therefore the “pre-flood years” in Alabama coincided with “post oil spill” UME years, where stranding events were already elevated. This also explains why the number of strandings in Alabama were found to be statistically higher overall than either the Florida-impacted or non-impacted regions analyzed here. Second, the “non-flood-impacted” reference counties exhibited a substantial spike in stranding events in a single month of January 2016 (Fig 7), much higher than any reports for any region in 2014. A spike in the number of standings in the control group at any point could influence the power to detect significant increases between either treatment group and this reference group.

### Caveats and considerations

The potential impacts on our results due to photo quality and analytical caveats should be considered. The initial purpose of our photographic field efforts was to capture high quality dorsal fin photographs, so there are many cases of sighted individuals that were not photographed with enough body showing to be included in the dataset. The emphasis of our field effort shifted over time when the value of taking additional body photos was realized, but this was not until after noticing skin lesions on dolphins in the field, following the flood. Furthermore, early in our mark-recapture survey work, we used an older camera that we had some light sensor issues with, resulting in a lot of photos that were too dark for evaluation of skin lesions. Our quality control screening process was necessarily rigorous but also resulted in the removal of potentially valuable examples. For example, 39 dolphins were identified with skin lesions during the summer 2014 season but due to photo quality, available dolphin surface area, degree of uncertainty, or other artifacts of the images, only 18 were included in the final dataset for this season. Despite the data loss, we are confident in the resulting dataset and the positive cases that were identified, acknowledging that results are likely conservative. This was preferred over including uncertain cases, which would likely have exaggerated our interpretation of the flood impacts.

The nature of the dataset made formal analyses challenging. The low numbers of dolphins per season that demonstrated skin lesions combined with unequal sample sizes across seasons, and inconsistencies in resightings of individuals over time, resulted in a dataset with low power for many analytical approaches. Data were combined across the three post-seasons for comparison to the pre-flood winter season, which functioned to increase the sample sizes available for analyses; however, it also likely biased results in favor of finding a significant change in prevalence or extent, simply because any given individual had three times the opportunity to be included in the post-flood dataset compared to the single pre-flood season dataset (and if seen in all three post-flood seasons, would also have three times the number of photos available from which to detect skin lesions).

The utility of quantitatively evaluating specific lesion categories of unknown etiology is questionable, given the varied lesions found in different regions, species, and the wide variety of descriptors and terms used by researchers among studies [37]. Therefore, we did not analyze changes in prevalence with respect to specific categories in this study because we did not feel that it added value. Although the category groupings here were found to be more reliable than the initial approach [37], without samples from which to evaluate etiologies, these categories are necessarily descriptive. Our photos were initially reviewed by veterinarians and pathologists with more lesion experience to help guide our detection process and teach us important visual cues. However, there was still very little known at the time about how freshwater impacted dolphins, and therefore limited available information on how to visually identify skin conditions of importance from photos alone, beyond obvious cases.

It’s possible that many lesions scored as *PP* were in fact freshwater lesions without pathogens present. The properties that define freshwater exposure-related skin lesions are still poorly understood, and with only photos to go by, it was impossible to clearly distinguish “freshwater lesions” from non-freshwater associated lesions. Six years after the flood, additional cases from around the NGoM are becoming available to facilitate a better understanding of these occurrences and events. Pathophysiological and biochemistry data from more recent out-of-habitat cases have started to inform what data should be collected and analyzed [23, 29; also the topic of discussion (i.e. unpublished case studies) at a NOAA-led workshop hosted in St. Petersburg, Florida, July 28–29, 2019, attended by the first author]. These additional cases have also helped to identify some common gross trends for what happens to skin in response to freshwater exposure. One visual cue that has been more broadly associated with freshwater exposure is skin pallor, where dolphins exhibit an overall paleness. This was not a visual cue included here since we did not have this understanding at the time of assessment, but in retrospect, it appears to be a condition that was present after the flood. Prevalence estimates presented here are therefore likely lower than they would be with this characteristic in mind.

Several biases may have been introduced as a result of the varying field effort in fall of 2014. Fewer days on the water to ‘capture’ and ‘recapture’ individuals may have resulted in decreased capture probabilities (i.e. fewer opportunities to sight individuals present and fewer total dolphins detected), which could have artificially inflated prevalence estimates if the number of skin lesioned animals remained stable from summer to fall. Capture probabilities for that season were very low (*p* = 0.09) but identical to the summer 2014 season (*p* = 0.09), and comparably low compared to the previous fall mark-recapture season (*p* = 0.14; based on data presented elsewhere: [40]). Raw counts of dolphins with lesions were highest for fall 2014 (Table 1), despite fewer surveys days. Fewer days on the water may have also resulted in a reduction in the number of images available per dolphin from which to assess skin lesions for that season, thereby biasing skin lesion extent estimates. However, the average number of photos taken per dolphin was highest in the fall of 2014 (13.3) compared to the other seasons (winter 2013–2014 = 9.4; spring 2014 = 9.0, summer 2014 = 9.8). Data collapsed from surveys over a two-month time span could artificially inflate lesion extent estimates for this season since there was more time for lesions to develop compared to the two-week survey windows from other seasons. To evaluate this possibility, the number of dolphins observed with lesions in October and respective extent ratings were compared to those observed in December of that season. Of the 127 total dolphins observed in fall of 2014, 105 (83%) of them were seen in October (20 of these dolphins were observed with skin lesions) compared to 30 (24%) observed for the first time in December (10 of these were observed with skin lesions). Of the dolphins seen this season with lesion extent scores above background levels, 13 of them were seen in October (four of these received moderate extent scores) compared to six seen for the first time in December (three of these received moderate extent scores). There were more dolphins seen in October overall, more with skin lesions detected, and more with higher levels of extent compared to those detected in December, but we acknowledge that there may still be introduced bias from those December cases. We have no way of knowing if these December cases would have been similarly scored in October, however, out of four skin lesioned dolphins seen in both months, one dolphin scored with moderate skin lesion extent in October was scored with background levels by December (a large hyperpigmented patch on the peduncle was no longer present) and the extent ratings for the remaining three dolphins was unchanged. If we removed the six dolphins only seen in December that were scored with greater than background skin lesion extent scores, the fall 2014 totals in Table 3 for low extent ratings would change from 12 to nine, and totals for moderate extent would reduce from seven to four. Despite fewer surveys days and an extended fall 2014 sampling season, the combined evidence does not change our overall conclusion that prevalence and extent measures were highest in the fall 2014 season.

Finally, given that we found background levels of skin lesions present prior to the flood, natural seasonal variation in temperature and/or freshwater input could be a confounding factor when considering the increase in skin lesions over the time period presented. However, the impetus for this project was the observed lesions following the flood event, which had not been observed in previous seasons of data collection (including the fall and summer of 2013). We confirmed this by reviewing the prior seasons of data. After accounting for photo quality, only nine dolphins were observed with skin lesions in the summer of 2013 and six were observed in fall 2013 (data not presented here).

### Skin lesions

The raw counts and calculated prevalence of skin lesions increased following the flood and the number of individuals with higher levels of lesion extent also increased, but not by numbers one might expect in response to such a drastic and immediate change in the environment. There was a significant relationship between season and lesion extent, but only with respect to the fall season following the flood. Very few individuals were seen with lesion extent >20% in any season (*n* = 11). There were low levels of skin lesion issues already present on the local dolphins, which had been previously unreported. This is perhaps not surprising given the subtlety of lesions found in the winter 2013–2014 season and the fact that the focus of the field work leading up to spring 2014 had been on capturing the best dorsal images for mark-recapture estimates of abundance, not on capturing good quality images of entire bodies.

Despite the photographic and analytical challenges, the results clearly show that instead of a hypothesized skin lesion “outbreak” in response to the flood, a handful of individuals exhibited extensive skin lesions, and some of these cases persisted or worsened over time. This result was surprising given the assumption that dolphins were likely exposed to full freshwater for sustained periods. It’s possible that we missed an initial acute response, with lesions that were more extensive than observed subsequently, since we were not in the field until six days after the flood (and not into East or Escambia Bay systems until seven and eight days following, respectively). Dolphins can exhibit dramatic changes to epidermis cells relatively quickly following freshwater exposure that can then shed when returned to saltwater [65]. However, station P05 in Figs 5 and 6 marks the station that is located at the south end of Escambia (where it opens into Pensacola Bay) and it’s clear that the entirety of Escambia Bay was fresh (≤5 ppt) for at least a month and in July 2014, was ≤10 ppt in the south end while the northern section of the bay was still ≤5 ppt. As shown in Fig 2, sightings still occurred in upper Escambia Bay after the flood, demonstrating that dolphins were still utilizing this freshwater habitat. With the assumption that East Bay was also largely fresh over a similar time period, it’s unlikely that we missed a response that would have substantially changed by the time we were in the field.

Several other hypotheses could help explain our results. Given what is known about the general water quality of the system, freshwater alone may not have been the primary cause of skin lesions; a weakened immune system from freshwater exposure may have left dolphins more susceptible to other infectious pathogens and secondary infections. Similarly, a limited number of individuals may have had compromised immune systems before the flood and therefore have been more susceptible to viruses or other infectious pathogens dumped into the system during the flood. The fact that we saw dolphins with lesions during the winter of 2013–2014 supports this as a possibility. 18 dolphins (13%) were seen prior to the flood with lesions (rater confidence moderate to high but including five that didn’t pass photo screening). Eleven of those were seen again following the flood. Six of these exhibited increased lesion extent in at least one season following the flood (and are included in Table 4) and comprised 18% of all individuals seen with lesion extent ≥5% at least once over the course of the study. Furthermore, about half of the dolphins that were seen with lesion extent above 5% did not demonstrate extent at this level or higher until the fall following the flood (6–9 months later and several months after the freshwater lens was gone). A case study is discussed in Toms et al. [40] in which an individual with more extensive skin lesions was seen in freshwater conditions in the upper Escambia Bay system following the flood. In photos taken before the flood, however, the individual was also heavily raked and was already showing a light degree of skin pallor. This dolphin may have already been in less-than-optimal health before the storm and therefore more susceptible to stressors associated with the flood that led to the development of the extensive lesions seen two weeks after the flood. The relationships between freshwater exposure and the physiological and biochemical responses of dolphins are still poorly understood but accumulating case studies suggest that rapid changes can occur within hours of freshwater exposure [23, 29, 34]. The potentially delayed effect that we observed, with increasing lesion extent months following the flood, is further suggestive that freshwater exposure may not have been the only, or even primary, factor involved in lesion expression for the time period evaluated.

Another hypothesis for why lesion prevalence and extent were not more widespread is that dolphins have somewhat adapted locally to natural fluctuations in the environment and are more tolerant physiologically and less susceptible to the immediate impacts of freshwater alone. Hagy and Murrell [13] described water flow in the Pensacola Bay system under low, moderate, and high freshwater inflow conditions. During their study period (2002–2004), extremely high inflow rates were shown to completely displace saltwater from Escambia and Blackwater Bay. The authors also mention that the long-term records showed periods of very high flow occurred once every few years, in which case it may not be uncommon for large sections of these northern estuary systems to be inundated with freshwater. Recent work in Barataria Bay, Louisiana demonstrated that dolphins could tolerate salinities <10 ppt in that system without showing skin lesions and may be more tolerant to a lower salinity habitat [33]. Ten dolphins were seen in the Escambia Bay system when it was nearly completely fresh in the spring of 2014. Eight of the ten had confirmed lesions (the other two were neonates and photos did not show their bodies). Interestingly, all of them were frequently sighted in low salinity areas, outside of the flood-impacted time frame, and yet did not exhibit chronic lesions indicative of freshwater [40]. Alternatively, dolphins may adapt simply by shifting habitat use to areas with increased salinity levels (e.g. towards deep channels or the southern end of the system) or by capitalizing on stratified water columns with increased salinities at depth in order to mitigate the effects of freshwater exposure. In the Barataria Bay study, tracking data revealed that most dolphins leave low salinity (<10 ppt) conditions within 24 hours, and very few enter waters with salinities <5 ppt [33], suggesting that dolphins may actively shift out of low salinity waters before it becomes detrimental to their health. Past work in the neighboring Choctawhatchee Bay system showed that relative dolphin abundance in the system was significantly lower in a year associated with extensive flooding and lower annual salinities in the bay system [66], suggesting that many left the system in response to the flood.

Conversely, however, there are case studies that report dolphins seemingly choosing to stay in freshwater habitats for prolonged periods of time, even when detrimental to their health. For example, Mullin et al. [30] reported a community of bottlenose dolphins that were monitored in Lake Pontchartrain for four years. 90% of them exhibited skin lesions, many of which were much more extensive than those described here. Skin lesions were reported to worsen with lower salinities, and it’s expected that their disappearance was due to death (given the increase in mortality events in the area associated with the disappearance of live animals detected in the system, although not confirmed). It was initially suggested that these dolphins were trapped in the freshwater habitat after being displaced by Hurricane Katrina, yet new dolphins were seen in the area over time, suggesting that they could move in and out. Another case study in this report described a single male dolphin observed regularly for three years in the canals of a housing development off Lake Pontchartrain (also very fresh water). He was last reported with skin lesions covering ~50% of body and then found dead 12 days later. Perhaps the dolphins that live in inshore habitats that regularly experience freshwater (e.g. Barataria Bay and Pensacola Bay) are able to respond in ways to mitigate effects of exposure, whereas dolphins that find themselves displaced into entire new habitats (e.g. Lake Pontchartrain region), or are not regularly exposed to freshwater conditions, do not have the experience to know how to mitigate exposure.

Our results are based only on data obtained from individuals that remained in the system, were seen in the months following the flood, and survived. What remains unknown was how many individuals may have left the system in response to poor conditions or remained in the bay but were not detected. For example, catalog IDs 664 and 665 were a mom and calf pair that were seen 19 days after the flood and exhibited the worst skin lesions recorded across the entire study period (the mom was the only dolphin to be rated with an extent of 4, and 74% lesion extent was calculated from a trace of the visible skin area) but they were never seen again. The stranding data offered some insight on dolphins that may have gone undetected. Regardless of the analytical challenges already discussed, there was an increase in stranding events (including perinates) in both Alabama and Florida’s flood-impacted region right after the flood. It’s unclear if this can be attributed specifically to the flood event or if it’s simply a part of the natural peak in stranding events, historically typical in the NGoM during this time of year, coinciding with calving season [56, 60, 67]. At the time of the flood, there were no specific protocols in place to evaluate freshwater exposure symptoms in stranded dolphins. Protocols are currently in development but the notes of freshwater exposure at the time varied by region and were primarily anecdotal. Even so, there were four animals that stranded in Alabama that were all reported with lesions associated with freshwater exposure from April 20 –May 25, 2014, and three of these were following the flood.

## Conclusions

The research presented here is a necessary step in the incremental development of the ability to characterize freshwater effects and impacts. We were uniquely positioned with a baseline available from which to evaluate the response to an extreme weather event. There’s growing evidence that freshwater exposure is detrimental to dolphin health and the scientific community is just starting to understand why and under what conditions. The results from the Pensacola event highlight how little we understand. Despite an extreme 100-year flood, dolphins in the area do not appear to have been widely affected by the flood alone. Photo ID work across the entire mark-recapture study was not complete at the time of this publication but from what was known, of the 32 dolphins that were seen with lesion extent ≥5% at some point in the four seasons surrounding the flood, at least 22 (~69%) were still alive at least a year following the flood. The factors that determine whether flooding and freshwater exposure will lead to serious health concerns or result in mild responses are still poorly understood, and the accumulation of reports such as this will help move the needle forward. Many dolphins remained in the area despite low salinities and potentially poor habitat quality which has huge implications for how we manage populations that may be at higher risk to freshwater intrusion. There is a proposed Mid-Barataria Sediment Diversion project in progress at the time of this publication (https://coastal.la.gov/midbarataria/), which would introduce intermittently released freshwater into the Barataria Bay system, home to a different population of bottlenose dolphins in Louisiana. Accumulating reports from natural events such as the one reported here have been informative for the developing mitigation measures in Louisiana (https://www.mmc.gov/events-meetings-and-workshops/other-events/effects-of-low-salinity-exposure-on-bottlenose-dolphins-webinar). We offer new hypotheses to help explain why dolphins may or may not face high health risks with such exposure. A better understanding of risk factors and/or mitigation solutions will continue to be especially important in areas like the NGoM where the frequency and intensity of storms are expected to increase over time, in the face of global climate change.

## Supporting information

S1 File Updated criteria for evaluating skin lesion categories. (DOCX)

S2 File Additional stranding data. (DOCX)

S3 File Rainfall displacement calculations. (DOCX)

S4 File Lesion extent data. (XLSX)

S5 File Lesion prevalence data. (XLSX)
