# Supplementary material for: Skin lesion and mortality rate estimates for common bottlenose dolphin (Tursiops truncatus) in the Florida Panhandle following a historic flood
Source: PLoS One. 2021 Oct 7;16(10):e0257526. doi: 10.1371/journal.pone.0257526 (PMC8496785; doi:10.1371/journal.pone.0257526)
Supplement: S1 File — (DOCX) [file pone.0257526.s001.docx]

The following supplements accompany the article

**Potential consequences of a historic flood on common bottlenose dolphin (*Tursiops truncatus*) skin lesions and mortality rates in the Florida Panhandle.**

Christina N. Toms*, Tori Stone, Traci Och

*Corresponding Author: ctoms@mote.org

## S1 Supporting Information

### **Updated Criteria for Evaluating Skin Lesion Categories**

Toms et al. [1] demonstrated that independent raters struggled to achieve acceptable levels of reliability when there were many categories to distinguish among and when soring lesions in the form of small spots (e.g., black spots, fringed, or cloudy white spots). Given that skin lesions were grouped here simply for descriptive and summary purposes, only six categories were used, reduced from the original 17 previously described. Without samples from which to test etiologies, these groupings were expected to be more meaningful in summaries. In context of the flood event, potentially pathogenic lesions were of primary interest, given the historic water quality issues in the Pensacola Bay system. Therefore, based on the literature reviewed in Toms et al. [1], the following lesions were grouped as potentially pathogenic (PP): tattoo, lunar, dark fringe, white fringe, cloudy white spots, spotted, vesicular, and dark spot lesions. These lesions were categorized only if they were seen on areas of the body without rake marks. Otherwise, they were instead categorized as rake mark-associated potentially pathogenic (RMA-PP). Orange lesions combined previously described orange hue and orange patches. Hypopigmentation included the originally delineated white amorphous lesion type and mottled type if they were primarily light in pigmentation. Hyperpigmentation included mottled lesions if they were primarily dark in pigmentation. The discolored head and/or nuchal patch category was retained from Toms et al. [1] but included instead as a sub-category here of hypo- or hyper­pigmentation, since the only delineating factor was the location of the discoloration. Therefore, it was also scored positively for hypopigmentation or hyperpigmentation depending on which pigmentation type was more prominent.

Results from Toms et al. [1] suggested that the operational definitions of lesions in the form of small spots could be improved upon to increase interrater reliability and biological relevancy. Recommendations included increasing the minimum number and/or size criteria inclusion. Therefore, criteria on how to handle these types of skin lesions were refined here and interrater reliability re-assessed. We focused on lesion spots that had the potential to be pathogenic (cloudy white spots, dark black spots, black fringe spots, white fringe spots, and vesicular lesions), given links reported in the literature [elaborated on by: 1]. Individual dolphins scored with only spot-types of lesions present were retained under the following three criteria:

- Minimum size and number criteria: if there was a minimum of three spots (did not have to all be the same variety) of a size greater than ~2 cm in diameter (approximately the size of a quarter, roughly estimated using examples in a key since we had no way to standardize this) and all three spots were given a rater certainty score of moderate or better and photo quality standards (see main text, methods section) were met for all three spots.
- Minimum size criteria: if there were less than three spots but at least one of the spots was larger than 2 cm and certainty was high and photo quality standards were met.
- Black spots criteria: if black spots were smaller than 2 cm but there were more than three of them and they were dark and pitted, and certainty was moderate or better and photo quality standards were met. Hyperpigmentation spots were excluded if they were not dark in saturation (*e.g.,* grey spots were excluded).

#### **Reassessment of Reliability**

Our updated approach to screening data and scoring skin lesions called for a reassessment of the reliability of our approach. Two raters (with past experience evaluating skin lesions) scored 50% of the data independently (*n* = 269). Reliability was assessed for the scores of presence and absence and for the decision to retain an individual for analyses, given our updated screening process. Data were analyzed using the “epi.kappa” function in R package, “epiR” [2], to estimate Cohen’s Kappa [3] and tested for significance using a *z* – test (*Ho*: *K* = 0.40; if 0.40 fell below the lower confidence interval, K was considered significantly greater than 0.40) [4]. Additional metrics were also calculated, as described in Toms et al. [1]: prevalence effects, bias effects, PABAK, and *K_max_* [also see: 4, 5-7]. Bias effects were tested for significance using a McNemar test [6]. Raters showed significant levels of reliability (*p* < 0.05, Table S1.1) with high percent agreement both for scoring the presence/absence of skin lesions (*K* = 0.60) and for deciding whether or not to retain individuals for analyses (*K* = 0.70). Kappa values (*K*) ranging from 0.60 – 0.70 were interpreted as reasonable estimates of reliability if there were substantial prevalence and/or bias effects impacting the analyses. The winter dataset had few individuals with lesions, which reduced the opportunity for raters to provide a positive score, thereby increasing the probability of chance agreement and decreasing calculated levels of *K.*

**Table S1.1:** Reliability results for scoring the presence and absence of skin lesions and on the decision to retain data for analyses.

| Lesion Type | Cohen's Kappa Value (*K*) | *z*-test statistic^^^ | *z*-test SE (CI) | Percent Agreement | Probability of Change Agreement | Prevalence Index (PI) | Bias Index (BI)^^^^ | PABAK (Corrected Kappa) | *K_max_* | Reliability Quality |
| --- | --- | --- | --- | --- | --- | --- | --- | --- | --- | --- |
| Winter ’13-‘14  (retain data) | 0.55 | 4.91 | 0.11  (0.33-0.77) | 90.6% | 0.79 | 0.77 | 0.07** | 0.81 | 0.84 | Poor-moderate Heavy bias/ prevalence/chance |
| Winter ’13-‘14  (presence /absence) | 0.55 | 5.03 | 0.10  (0.33-0.71) | 79.8% | 0.58 | 0.45 | 0.18 | 0.60 | 0.62 | Poor- heavy bias |
| Spring 2014  (retain data) | 0.82 | 12.7* | 0.07 (0.68-0.95) | 93.5% | 0.65 | 0.55 | 0.01 | 0.87 | 0.97 | Excellent |
| Spring 2014  (presence /absence) | 0.66 | 9.31* | 0.09 (0.49-0.83) | 82.4% | 0.49 | 0.04 | 0.15** | 0.65 | 0.71 | Moderate – heavy bias |
| Combined  (retain data) | 0.70 | 11.22* | 0.06 (0.57-0.82) | 91.8% | 0.73 | 0.68 | 0.04* | 0.84 | 0.86 | Good - heavy chance & prevalence/bias |
| Combined  (presence /absence) | 0.60 | 9.48* | 0.06 (0.48-0.72) | 81.0% | 0.51 | 0.22 | 0.15** | 0.61 | 0.69 | Moderate to Good given bias effect |

*Note:* 50% of the dataset were scored for reliability (winter and spring, 2014 seasons); Reliability was evaluated for each season separately and as a combined dataset; Reliability for winter data was reduced due do having only a few chances to score “present” for skin lesions detected in this season; CI: Confidence Interval; ^^^*z*-test statistic for kappa; *Ho*: *K* = 0.4; ^^^^Tested for significance using McNemar test (*Χ*^2^) for bias (McNemar, 1947); *K_max_*: maximum attainable kappa given the difference in the proportions of positive classifications between raters; All significance tested with *α* = 0.05 (*N* = 110); **p* < 0.05; ***p* < 0.01.

Reliability for scoring extent was re-assessed based on ratings from two raters who each scored 50% of the data (*n* = 38) independently, producing a rating dataset with choices scored as 1-4 (trace, low, moderate, or high lesion extent). Since there were four levels to the dataset instead of two, reliability coefficients were calculated using the “cohen.kappa” function in R package “psych” [8] to estimate a weighted Cohen’s Kappa. A weighted Kappa was used since the difference in how two raters scored something was less problematic if they differed in their choice by 1 degree (*e.g.,* rater one scored a 1 and rater two scored a 2) than if they differed by 2 or 3 degrees (*e.g.,* rater scored a 1 and rater two scored 3). Results indicated good reliability (weighted *K* = 0.85; percent agreement = 84.2%).

### **Comparing Rating vs. Tracing Data for Measuring Skin Lesion Extent**

In a previous review [1] we demonstrated that interrater reliability results were good for both measures of skin lesions extent tested (*i.e.*, either using a categorical rating system or by tracing skin lesions using ImageJ) but did not assess how these two measures differed in terms of analytical utility or power to guide data interpretation. We address that here.

#### **Methods**

Categorical ratings were measured using the method described in text. Extent tracings were completed as described in Toms et al. [1]. Extent measures were an average of tracings of the left and right sides, when available. Proportions were calculated separately for both the dorsal only and the dorsal fin and visible body combined (referred to herein as dorsal fin + body). The former was included to provide a standardized comparison of extent across seasons, given that the dorsal fin could be measured consistently across individuals and time. However, measures from the dorsal fin alone were shown to be a poor proxy of extent measures of the dorsal fin + body combined, when analyzing photo data [1], such that it should not be interpreted as the sole measure of lesion extent without risking substantial bias in interpretation. Conversely, measures of the dorsal fin + body cannot be standardized since dolphins expose varying amounts of body at every surfacing and depending on their behavior. Congruency of results using both measures would help to increase confidence in interpretation, despite these limitations. If both sides of the animal were available, then both were traced and extent was calculated as an average of the two sides. Otherwise, tracings were completed for the single best photo of the individual.

#### **Results**

Estimates for lesion extent are summarized in Table S1.2 by season and estimate type. Lesion extent results were similar among the different measures used but ratings and the tracings of dorsal fin + body agreed more often. Dorsal fin tracings of extent agreed with dorsal fin + body traces in 38 of 54 cases (70%) but with ratings in only 34 of 64 cases (53%). Dorsal fin + body traces matched ratings in 49 of 57 cases (86%). Where dorsal traces disagreed with the other two measures, it was more commonly by underestimating extent. When compared with ratings, there were 26 cases where the dorsal trace underestimated the extent vs. only two cases where dorsal traces were higher than ratings. When compared to dorsal fin + body traces, the dorsal traces underestimated 15 times and only overestimated once. In cases where the ratings differed from dorsal fin + body trace results, ratings were higher as a result of capturing additional lesion types that could not be traced (e.g., orange hue, mottling, freckling).

**Table S1.2:** Side by side comparison of skin lesion extent measured as categorical ratings and as traces of images.

|  | Lesion Extent Rating | | | | | | Lesion Extent Tracing | | |
| --- | --- | --- | --- | --- | --- | --- | --- | --- | --- |
|  | 1 | 2 | 3 | 4 | Total | Avg. Dorsal Extent (range) | | Avg. Dorsal+Body Extent (range) |  |
| Winter ’13-‘14 | 12 | 1 | 0 | 0 | 13 | 0.01 (0-0.06) | | 0.01 (0.0003- 0.08) |  |
| Spring 2014 | 16 | 5 | 3 | 1 | 25 | 0.06 (0-0.47) | | 0.09 (0.0004-0.74) |  |
| Summer 2014 | 10 | 5 | 0 | 0 | 15 | 0.02 (0-0.16) | | 0.03 (0.001-0.16) |  |
| Fall 2014 | 10 | 12 | 7 | 0 | 29 | 0.02 (0-0.16) | | 0.04 (0.0003-0.30) |  |

*Note*: Lesion rating categories: 1 = background levels (<5% coverage of visible epidermis); 2 = low (5-20% coverage of visible epidermis); 3 = moderate (20-50% coverage of visible epidermis); 4 = high (>50% coverage of visible epidermis); ≥10% of the body had to be visible in photos in order to be scored for extent, using any of these measures.

As discussed in the main results section, there was a significant increase in the proportion of dolphins seen post-flood with lesion extent ≥5% compared to pre-flood (McNemar’s exact tests: *n* = 68, *p* = 0.001) when analyzed using the rating scores. Results when analyzed using tracing measurements of dorsal only or dorsal fin + body were not significant (McNemar’s exact tests with Bonferroni correction: *n* = 65, *n* = 61, respectively; *p*’s > 0.02) but suffered from low numbers of individuals that could be traced. We chose to report results based on rating data. It’s still possible that the visible proportion of dorsal + body biased our final rating measures. If so, it would be in a conservative direction given our results. Given the way dolphins surface, there were likely far greater number of cases with a smaller proportion of visible skin surface area compared to those where we saw a large proportion of the body visible. In which case, we risk overestimating lesion extent if there is a large lesion in that small area. Or we risk not detecting substantial skin lesions that are hidden underwater. Our results show that we had very few cases of moderate and high extent across the study period so it’s unlikely that we artificially increased our measures in a way that impacts our conclusions, in which case, the more likely scenario is that lesion extent was underrepresented.

## Supplemental Information References

1. Toms CN, Stone T, Och‐Adams T. Visual‐only assessments of skin lesions on free‐ranging common bottlenose dolphins (*Tursiops truncatus*): Reliability and utility of quantitative tools. Marine Mammal Science. 2020:1-30.

2. Stevenson M. Tools for the analysis of epidemiological data. R package version 0.9-97; 2018.

3. Cohen J. A coefficient of agreement for nominal scales. Educational and Psychological Measurement. 1960;20(1):37-46.

4. Sim J, Wright CC. The Kappa statistic in reliability studies: Use, interpretation, and sample size requirements. Physical Therapy. 2005;85(3):257-268.

5. Byrt T, Bishop J, Carlin JB. Bias, prevalence and kappa. Journal of Clinical Epidemiology. 1993;46(5):423-429.

6. Watson PF, Petrie A. Method agreement analysis: A review of correct methodology. Theriogenology. 2010;73(9):1167-1179.

7. Altman DG, Machin D, Bryant TN, Gardner MJ. Statistics with confidence: Confidence intervals and statistical guidelines. 2nd edition ed. London: GMJ Books; 2000. 254 p.

8. Revelle W. psych: Procedures for personality and psychological research. Version = 1.8.12 ed. Evanston, Illinois, USA: Northwestern University; 2018.
