# Supplementary material for: Skin lesion and mortality rate estimates for common bottlenose dolphin (Tursiops truncatus) in the Florida Panhandle following a historic flood
Source: PLoS One. 2021 Oct 7;16(10):e0257526. doi: 10.1371/journal.pone.0257526 (PMC8496785; doi:10.1371/journal.pone.0257526)
Supplement: S2 File — (DOCX) [file pone.0257526.s002.docx]

The following supplements accompany the article

**Potential consequences of a historic flood on common bottlenose dolphin (*Tursiops truncatus*) skin lesions and mortality rates in the Florida Panhandle.**

Christina N. Toms*, Tori Stone, Traci Och

*Corresponding Author: ctoms@mote.org

.

## S2 Supporting Information

### **Additional Stranding Data**

**Table S2.1:** Number of total cetaceans and *Tursiops truncatus* (*Tt*) only, stranded per year for the different geographic ranges considered.

|  | 2012 | | | | 2013 | | | | 2014 | | | | 2015 | | | | 2016 | | | | Overall | | | |
| --- | --- | --- | --- | --- | --- | --- | --- | --- | --- | --- | --- | --- | --- | --- | --- | --- | --- | --- | --- | --- | --- | --- | --- | --- |
|  | TOT | # Dead | % Dead | # Perinate | TOT | # Dead | % Dead | # Perinate | TOT | # Dead | % Dead | # Perinate | TOT | # Dead | % Dead | # Perinate | TOT | # Dead | % Dead | # Perinate | TOT | Tot Dead | % Dead | Tot Perinates |
| Cetaceans (all) | 59 | 58 | 98% | n/a | 53 | 51 | 96% | n/a | 87 | 85 | 98% | n/a | 72 | 70 | 97% | n/a | 102 | 98 | 96% | n/a | 373 | 362 | 97% | n/a |
| Cetaceans Panhandle only | 29 | 29 | 100% | n/a | 26 | 25 | 96% | n/a | 47 | 45 | 96% | n/a | 45 | 43 | 96% | n/a | 51 | 51 | 100% | n/a | 198 | 193 | 97% | n/a |
| *Tt* only (all regions) | 47 | 46 | 98% | 13 | 43 | 42 | 98% | 11 | 70 | 68 | 97% | 20 | 61 | 60 | 98% | 15 | 78 | 76 | 97% | 9 | 299 | 292 | 98% | 68 |
| *Tt* - all FL Panhandle | 20 | 20 | 100% | 6 | 19 | 19 | 100% | 5 | 37 | 35 | 95% | 10 | 37 | 36 | 97% | 7 | 42 | 42 | 100% | 3 | 155 | 152 | 98% | 31 |
| *Tt* - AL only | 27 | 26 | 96% | 7 | 24 | 23 | 96% | 6 | 33 | 33 | 100% | 10 | 24 | 24 | 100% | 8 | 36 | 34 | 94% | 6 | 144 | 140 | 97% | 37 |
| *Tt* - FL Flood-Impacted | 10 | 10 | 100% | 5 | 7 | 7 | 100% | 2 | 20 | 18 | 90% | 7 | 14 | 13 | 93% | 3 | 16 | 16 | 100% | 2 | 67 | 64 | 96% | 19 |
| *Tt* - FL Non-Impacted | 10 | 10 | 100% | 1 | 12 | 12 | 100% | 3 | 17 | 17 | 100% | 3 | 23 | 23 | 100% | 4 | 26 | 26 | 100% | 1 | 88 | 88 | 100% | 12 |

*Note: Tt*: *Tursiops truncatus*; Panhandle here is defined as the geographic range from the Alabama-Florida border to Franklin County, Florida. Florida flood-impacted counties included: Escambia, Okaloosa, and Santa Rosa counties; Non-flood-impacted counties in the Florida Panhandle included: Bay, Franklin, Gulf, Wakulla, and Walton counties; Perinates defined as any full carcass with a length of <115 cm.


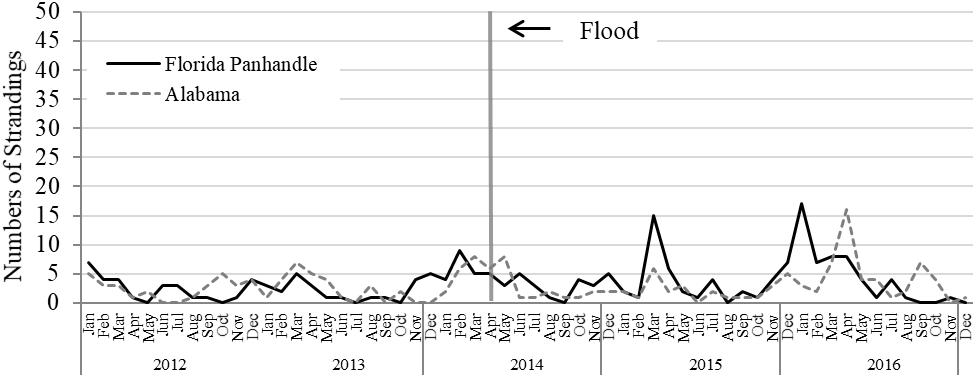


All Cetaceans


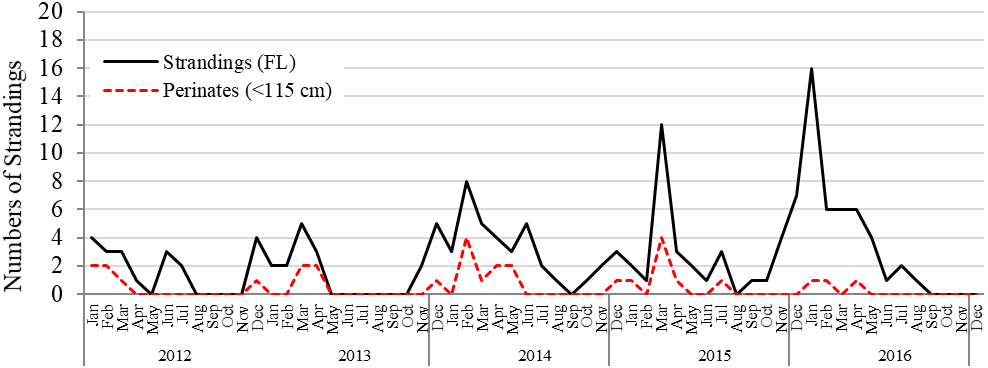


*Tt* only – All of Florida Panhandle

Flood

**Fig S2.1: Total number of individuals that stranded per month from 2012-2016 in the Northern Gulf of Mexico**; Top: strandings of all cetaceans in Alabama and the Florida Panhandle, scaled to match Litz et al. [1] for direct comparison; Bottom: all strandings of only *T. truncatus* across both flood-impacted and non-impacted regions of the Florida Panhandle combined; Florida Panhandle = Alabama-Florida border to Franklin County, Florida.

## Supplemental Information References

1. Litz JA, Baran MA, Bowen-Stevens SR, Carmichael RH, Colegrove KM, Garrison LP, et al. Review of historical unusual mortality events (UMEs) in the Gulf of Mexico (1990-2009): Providing context for the multi-year northern Gulf of Mexico cetacean UME declared in 2010. Diseases of Aquatic Organisms. 2014;112(2):161-175.
