# Supplementary material for: Skin lesion and mortality rate estimates for common bottlenose dolphin (Tursiops truncatus) in the Florida Panhandle following a historic flood
Source: PLoS One. 2021 Oct 7;16(10):e0257526. doi: 10.1371/journal.pone.0257526 (PMC8496785; doi:10.1371/journal.pone.0257526)
Supplement: S3 File — (DOCX) [file pone.0257526.s003.docx]

The following supplements accompany the article

**Potential consequences of a historic flood on common bottlenose dolphin (*Tursiops truncatus*) skin lesions and mortality rates in the Florida Panhandle.**

Christina N. Toms*, Tori Stone, Traci Och

*Corresponding Author: ctoms@mote.org

## S3 Supporting Information

### **Rainfall Displacement Calculations**

To estimate the freshwater input into the system from the flood event, freshwater inputs (Q) were calculated (below) for three different input sources: ungauged freshwater flow from the watershed, gauged freshwater flow from the watershed, and direct input from rainfall. Ungauged and gauged watershed areas, bay surface area, and bay volume (Table S3.1) were reported in Hagy and Murrell [1]. Daily freshwater discharge from four gauging flow stations (site ID) were obtained from the USGS (<https://nwis.waterdata.usgs.gov/nwis/dv>): Escambia River at Molino (02376033), Blackwater River (02370000), Big Coldwater Creek (02370500), and Yellow River (02368000). Daily data were summed across stations spanning from April 1 – May 31, 2014. Prior to the first rain event in April, daily discharge rates averaged 43E+06 m^3^/d, which we considered to be a baseline flow. Flows returned to baseline levels on May 5, 2014 (Fig S3.1). Therefore, the event flow was calculated as the sum over six days of the increase in the gauged flow rate above base flow. This source accounted for about 12% of the volume of the bay. The rainfall to the lower ungauged watershed was assumed to be equal to the local rainfall around the Bay, or 25 inches (63.5 cm). Given the high rate of rainfall and the fact that the ground was previously saturated from a rain event 14 days earlier, it was assumed that runoff was equal to the rainfall, implying that infiltration and evapotranspiration were negligible. The flow from the ungauged watershed was therefore 63.5 cm multiplied by 4659 km2, which is 2.96E+09 m3 for the event and about 267% of the volume of the bay. Direct rainfall of 63.5 cm of rain to the Bay, which has a mean depth of 3 m (=300 cm), displaced 63.5 cm / 300 cm, or 21% of the Bay volume.


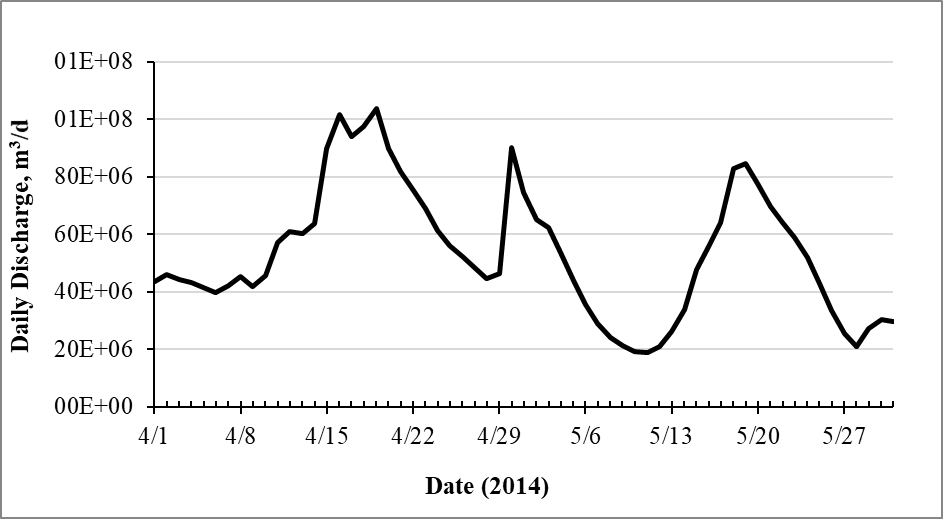


Flood rainfall started

**Fig S3.1.** **Total daily freshwater input from the gauged flow areas of the watershed.** Summed across four gauging flow stations from April 1 – May 31, 2014. The dotted line denotes an estimated baseline flow rate.

**Table S3.1.** **Flow rate calculations and bay volume**

| Freshwater Inflow from the Watersheds |  | Q (m^3^/event) |
| --- | --- | --- |
| Ungauged Area (4659 km^2^) |  | 4659E+06 m^2^ * 0.635 m = 2.96E+09 |
| Sum of Gauged Flows (13502 km^2^) |  | 1.34E+08 |
| Direct Freshwater Input |  |  |
| Bay Surface Area (370 km^2^) |  | 370E+06 m^2^ * 0.635 m = 2.35E+08 |
| Q Total (m^3^/event) |  | 3.33E+09 |
| Bay Volume |  | 1.11E+09 m3 |

## Supplemental Information References

1. Hagy JDI, Murrell MC. Susceptibility of a northern Gulf of Mexico estuary to hypoxia: An analysis using box models. Estuarine, Coastal and Shelf Science. 2007;74(1):239-253.
